# Is candidemia mortality inevitable or a consequence of inadequate guideline adherence?

**DOI:** 10.1016/j.bjid.2026.105824

**Published:** 2026-05-18

**Authors:** Murilo Freua Sequeira, Caio Trevelin Sambo, Bianca Leal de Almeida, Erika Yukie Ishigaki, Eloisa Basile Siqueira Ayub, Gabriel Fialkovitz, Adriana Satie Gonçalves Kono Magri, Olavo Henrique Munhoz Leite, Marcello Mihailenko Chaves Magri

**Affiliations:** a Centro Universitário FMABC, Disciplina de Infectologia, Santo André, SP, Brazil; b Universidade de São Paulo, Faculdade de Medicina, Hospital das Clínicas, (HCFMUSP), Divisão de Clínica de Moléstias Infecciosas e Parasitárias, São Paulo, SP, Brazil; c Universidade de São Paulo, Hospital das Clínicas, Instituto do Coração, Departamento de Infecção Hospitalar, São Paulo, SP, Brazil

**Keywords:** Candidemia, Source control, Central venous catheter, Care bundle, Infectious diseases consultation

## Abstract

**Background:**

Candidemia is a severe healthcare-associated infection with persistently high mortality, particularly in critically ill patients. Beyond host severity, inadequate implementation of recommended management strategies may substantially influence outcomes.

**Methods:**

This retrospective observational study included adult patients with at least one episode of candidemia in a Brazilian tertiary hospital between 2018 and 2024. Clinical, microbiological, and therapeutic variables were analyzed, including adherence to an eight-item candidemia care bundle. In-hospital mortality was the primary outcome. Univariate, multivariate logistic regression, and Kaplan-Meier survival analyses were performed.

**Results:**

Among 136 patients (mean age 62.4-years), 87.5% required ICU admission. Overall mortality reached 76.5%. *Candida albicans* (32.4%) and the *C. parapsilosis* complex (29.4%) were the most frequent species. Non-optimal treatment (OR = 5.52; p = 0.031) and failure to remove the central venous catheter (OR = 6.60; p = 0.004) were independent predictors of death. Catheter removal significantly improved survival (p < 0.001). Survivors showed greater adherence to the care bundle (3.66 vs. 1.98 items; p = 0.001) and more frequent Infectious Diseases consultation (50%vs. 28.8%; p = 0.027). Mortality did not differ between catheter-drawn and peripheral blood cultures.

**Conclusions:**

In this cohort, candidemia mortality was strongly associated with failure in early vascular source control and poor adherence to recommended management, underscoring quality of care as a key determinant of survival.

## Introduction

Candidemia is one of the most relevant healthcare-associated fungal infections in tertiary hospitals and is associated with high morbidity, mortality, and healthcare costs. Its transmission related to medical care and its high lethality reinforce the need for rigorous epidemiological surveillance and appropriate clinical management.^1^^,^^2^

Globally, approximately 626,081 cases of *Candida* spp. bloodstream infections occur annually, accounting for more than 85% of all fungemias in Europe and the United States.^3^ In Brazil, determining the true incidence of candidemia remains challenging due to limitations in diagnostic methods, shortage of specialized microbiology teams, and restricted access to effective antifungal therapy, factors that contribute to persistently elevated mortality rates. In a national study, among 7075 records analyzed, only 2305 contained detailed information regarding candidemia episodes, with *C. albicans, C. parapsilosis, and C. tropicalis* being the most prevalent species and a rising proportion of *C. glabrata*.^4^

Although the prescription of echinocandins has increased in the last decade, 30-day mortality rates have remained stagnant at high levels (50%–60%) in a Brazilian cohort conducted between 2010 and 2018. This scenario contrasts with European cohorts, such as those from Spain, where more assertive management characterized by early antifungal initiation and prompt central venous catheter removal has been associated with lower lethality.^5^ Similarly, Almeida et al. (2024)[6] demonstrated that inadequate management is an independent predictor of death, with lethality exceeding 60% in ICU patients and reaching 74.6% in oncological patients. These data reinforce that, beyond host severity, healthcare practices and management strategies play a central role in candidemia outcomes.

## Methods

This retrospective observational analytical study was conducted at Hospital Estadual Mário Covas, a 313-bed public tertiary university hospital located in Santo André, São Paulo, Brazil. The institution serves as a regional referral center for high-complexity care, including medical, surgical, and oncological patients, providing coverage to seven municipalities in the Greater ABC region. The patient population is characterized by a high burden of comorbidities, frequent need for invasive support, and prolonged hospitalizations. The study period extended from January 2018 to December 2024. Adult patients (≥ 18-years) hospitalized during this period who presented at least one episode of candidemia were included.

The study was approved by the Research Ethics Committee of Centro Universitário FMABC (CAAE 89,896,925.1.0000.0082). Due to the retrospective design, informed consent was waived. Data confidentiality followed Brazilian data protection law (LGPD, Law n° 13.709/2018) and the principles of the Declaration of Helsinki.

Candidemia was defined as the isolation of *Candida* spp. from blood cultures obtained by peripheral venipuncture and/or from invasive vascular catheters. Patients were excluded when clinical data were insufficient for analysis or when medical records were inaccessible.

Clinical and laboratory data were obtained from electronic medical records, microbiology laboratory reports, and the Infection Control Service database. Data collection was performed using REDCap (Research Electronic Data Capture) hosted at http://redcap.fmabc.br.

Demographic variables included age, sex assigned at birth, and race/ethnicity according to the Brazilian Institute of Geography and Statistics classification. Clinical variables included reason for hospitalization (clinical or surgical), ICU admission, comorbidities (cardiopathy, pneumopathy, chronic kidney disease, diabetes, immunosuppression, and oncological disease), body mass index classification, previous bacterial infection, prior *Candida* spp. colonization or infection, need for invasive mechanical ventilation, vasopressor use, hypotension defined as mean arterial pressure < 65 mmHg, need for renal replacement therapy, parenteral nutrition, and exclusive palliative care. Laboratory variables included anemia (hemoglobin < 12 g/dL), neutropenia (< 1000 cells/mm^3^), thrombocytopenia (< 150,000 cells/mm^3^), C-reactive protein values, and SOFA score when available. Microbiological variables comprised *Candida* spp. identification according to conventional hospital taxonomy, and antifungal susceptibility testing when performed.

Management-related variables included evaluation by the Infectious Diseases team (isolated consultation or shared follow-up), performance of candidemia staging tests (echocardiography, fundoscopy, and sequential blood cultures), identification of deep foci of infection, catheter removal and timing, antifungal therapy initiation, type and duration of antifungal treatment, empirical therapy, sequential therapy defined as echinocandin de-escalation to fluconazole after at least five days and negative control blood culture, and the definition of optimal treatment as the use of an echinocandin combined with catheter removal when present. Adherence to an eight-item candidemia care bundle was evaluated for each patient: fundoscopy, echocardiography, control blood cultures, central venous catheter removal, antifungal initiation within 72-hours from blood culture collection, Infectious Diseases consultation, sequential therapy, and investigation of deep focus. A bundle adherence score ranging from 0 to 8 was calculated and stratified into three categories (0-items, 1‒4 items, and 5‒8 items).

The primary outcome was in-hospital mortality, categorized as death within 15-days, between 15 and 30 days, and after 30-days from the candidemia episode. Continuous variables were expressed as means ± standard deviation or medians and interquartile ranges according to distribution, and categorical variables as absolute numbers and percentages. Unpaired Student’s *t*-test and Chi-Square test were used for univariate comparisons. Logistic regression (enter method) was applied to identify independent predictors of mortality, with variable selection based on clinical relevance and univariate significance due to the number of predictors relative to sample size. Comparisons among outcome groups were performed using one-way ANOVA or Kruskal-Wallis test when appropriate. Kaplan-Meier survival analysis was conducted, and curves were compared using the log-rank test according to catheter management. A two-tailed p-value < 0.05 was considered statistically significant. Statistical analyses were performed using JAMOVI version 2.3.24 (The Jamovi Project, Sydney, Australia).

## Results

**Fig. 1:**
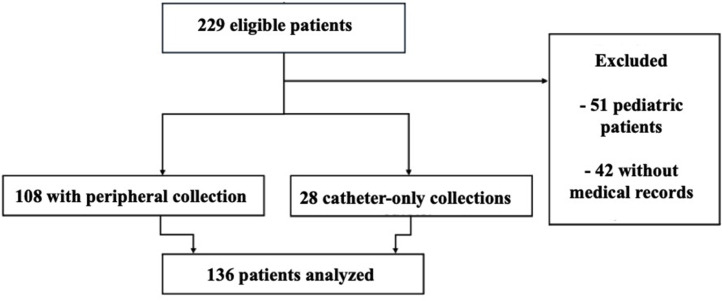
Study flowchart showing patient selection and final cohort composition.

Between January 2018 and December 2024, 136 adult patients with candidemia were included. Fig. 1 illustrates the study flowchart, detailing patient eligibility, exclusion criteria, and final cohort allocation for analysis. The cohort was predominantly female with 72 patients (52.9%), and 71 patients (52.2%) were classified as White. The mean age was 62.4-years (SD ±15.7). The mean length of hospital stay was 33.6-days (SD ±28). The mean interval between hospital admission and the first positive blood culture for *Candida* spp. was 18.8-days (SD ±18). The baseline characteristics of the study population are presented in Table 1.

**Table 1:** Baseline demographic, clinical characteristics, hospitalization features and laboratory findings of patients with candidemia (n = 136).

**Variable**	**n (%)**	**Mean ± SD**
Age (years)		62.4 ± 15.7
Length of hospital stay (days)		33.6 ± 28.0
**Sex**		
Female	72 (52.9)	
Male	64 (47.1)	
**Ethnicity**		
White	71 (52.2)	
Mixed	57 (41.9)	
Black	8 (5.9)	
**Comorbidities**		
None	12 (8.8)	
Heart disease	20 (14.7)	
Diabetes mellitus	45 (33.1)	
Pulmonary disease	7 (5.1)	
Chronic kidney disease	31 (22.8)	
Immunosuppression	50 (36.8)	
COVID-19	16 (11.8)	
Oncological disease	49 (36.0)	
**Body mass index**		
Obese	16 (11.8)	
Malnourished	24 (17.6)	
Not available	6 (4.4)	
**Hospitalization characteristics**		
ICU admission required	119 (87.5)	
Mean arterial pressure < 65 mmHg	91 (67.9)	
Vasopressor use	88 (62.5)	
Invasive mechanical ventilation	93 (68.9)	
Presence of vascular catheter	123 (90.4)	
Parenteral nutrition	18 (13.2)	
Renal replacement therapy during hospitalization	60 (44.1)	
Previous dialysis	23 (16.9)	
Infectious Diseases evaluation	46 (33.8)	
Surgical procedures during hospitalization	77 (56.6)	
Presumed bacterial infection	125 (91.9)	
Microbiologically confirmed bacterial infection	98 (72.1)	
Exclusive palliative care	21 (15.4)	
**Laboratory findings**		
Any hematological abnormality	130 (95.6)	
Neutropenia	17 (12.5)	
Thrombocytopenia	74 (54.4)	
Anemia	127 (93.4)	
SOFA score		8.47 ± 4.54
C-reactive protein (mg/dL)		20.1 ± 13.6

ICU, Intensive Care Unit; SD, Standard Deviation; SOFA, Sequential Organ Failure Assessment; MAP, Mean Arterial Pressure.

A total of 119 patients (87.5%) required intensive care unit admission during hospitalization. The first positive blood culture occurred in the ICU in 78 patients (57.3%), including 64 (47.1%) in the general ICU, 9 (6.6%) in the surgical ICU, and 5 (3.7%) in the coronary unit. First isolations also occurred in the emergency department in 11 patients (8.1%), in the clinical ward in 27 (19.9%), in the surgical ward in 15 (11.0%), and in the dialysis sector in 5 (3.7%).

Regarding comorbidities, 49 patients (36.0%) had a previous or active malignancy, 45 (33.0%) had diabetes mellitus, 20 (14.7%) had underlying cardiovascular disease, and 50 (36.7%) presented some form of immunosuppression. In terms of nutritional status, obesity was identified in 16 patients (11.7%) and malnutrition in 24 (17.6%), whereas most of the cohort (n = 90; 66.2%) was classified as eutrophic or overweight; nutritional data were unavailable in six cases (4.4%). Renal support was frequently required, with 60 patients (44.1%) undergoing renal replacement therapy during hospitalization, including 23 (16.9%) with pre-existing dialysis dependence.

Hypotension was observed in 91 patients (67.9%). Vasoactive drugs were required in 88 patients (62.5%), and invasive mechanical ventilation was necessary in 93 (68.9%). Broad-spectrum antibiotics were administered to 126 patients (94.0%). Presumed bacterial infection was recorded in 98 patients (72.1%), although microbiological confirmation was documented in 51 cases (37.5%). Vascular catheters were present in 123 patients (90.4%). Short-term central venous catheters were identified in 95 (70.4%), and temporary hemodialysis catheters in 43 (31.9%). Tunneled long-term dialysis catheters were present in 14 patients (10.4%), and totally implantable devices in 3 (2.2%).

*C. albicans* was the most frequently isolated species with 44 cases (32.3%). The *C. parapsilosis* complex was identified in 40 cases (29.4%), including *C. parapsilosis* in 29 (21.3%), *C. orthopsilosis* in 9 (6.6%), and *C. metapsilosis* in 2 (1.5%). *C. tropicalis* was isolated in 20 cases (14.7%) and *C. glabrata* in 18 (13.2%). Antifungal susceptibility testing was performed in 1 case (0.7%). The diagnostic evaluation, therapeutic management, and microbiological profile of candidemia are detailed in Table 2.

**Table 2:** Diagnostic practices, management strategies and *Candida* species profile in candidemia (n = 136).

**Variable**	**n (%)**	**Mean ± SD**
**Diagnostic work-up**		
Previous *Candida* colonization	30 (22.1)	
Fundoscopy performed	25 (18.4)	
Echocardiogram performed	49 (36.0)	
Abdominal CT scan	4 (2.9)	
Serial blood cultures performed	50 (36.8)	
Altered staging findings	9 (9.9)	
Deep focus identified	11 (10.8)	
Antifungal susceptibility testing performed	1 (0.7)	
Correct sequential blood cultures	17 (12.5)	
Persistent candidemia	6 (4.4)	
Previous history of *Candida* infection	5 (3.7)	
**Candidemia management**		
Treated for candidemia	70 (51.5)	
Empirical antifungal therapy	3 (4.2)	
Duration of antifungal therapy (days)		12.2 ± 14.8
Catheter removed	57 (47.9)	
Early catheter removal	39 (28.9)	
Late catheter removal	19 (14.1)	
Fluconazole	43 (31.6)	
Micafungin	48 (35.3)	
Amphotericin B deoxycholate	2 (1.4)	
Liposomal Amphotericin B	1 (1.7)	
Combination therapy	1 (1.4)	
Sequential therapy	9 (13.0)	
Optimal treatment	38 (27.9)	
**Bundle items performed**		2.38 ± 2.47
No bundle items	47 (34.6)	
1–4 bundle items	51 (37.5)	
5–8 bundle items	38 (27.9)	
***Candida* species identified**		
*Candida albicans*	44 (32.4)	
*Candida parapsilosis complex*	40 (29.4)	
*Candida metapsilosis*	2 (1.5)	
*Candida orthopsilosis*	9 (6.6)	
*Candida tropicalis*	20 (14.7)	
*Candida glabrata*	18 (13.2)	
*Candida dubliniensis*	4 (2.9)	
*Candida krusei*	3 (2.2)	
*Candida duobushaemulonii*	2 (1.4)	
Others	5 (3.7)	
Overall mortality	104 (76.5)	

CT, Computed Tomography; SD, Standard Deviation.

Antifungal therapy was initiated in 70 patients (51.5%). The mean treatment duration was 12.2-days (SD ±14.8). Micafungin was used in 48 treated patients (68.5%), and fluconazole in 43 (61.4%). Sequential therapy occurred in 9 treated patients (12.8%). Infectious Diseases consultation occurred in 46 patients (33.8%). Echocardiography was performed in 49 patients (36.0%), fundoscopy in 25 (18.4%), and follow-up blood cultures in 50 (36.8%). Persistent candidemia was documented in 6 patients (4.4%), however, this finding should be interpreted with caution given the limited performance of follow-up blood cultures in the cohort. A deep focus of infection was identified in 11 patients (8.0%). Catheter removal was performed in 57 patients (47.9%). The mean time to catheter removal was 5-days, and 39 removals (68.4%) occurred within the first 7-days.

Overall, in-hospital mortality reached 76.4% (104/136 patients). The majority of deaths occurred within the first 15-days following candidemia diagnosis (n = 75), whereas 11 patients died between 15- and 30-days (8.0%) and 18- after 30-days (13.2%). The mean time from diagnosis to death was 11.3-days (SD ±12).

In univariate analysis, patients who died were older than survivors (mean 64 vs. 57.3 years; p = 0.042). ICU admission was more frequent among those who died (93.3%vs. 68.8%; p < 0.001). Hypotension (78.6%vs. 32.2%; p < 0.001), vasopressor use (76.7%vs. 28.1%; p = 0.001), and invasive mechanical ventilation (79.8%vs. 32.2%; p < 0.001) were also more common in this group (Table 3). In contrast, catheter removal was significantly less frequent among patients who died (38.3%vs. 84.0%; p < 0.001). Previous chronic kidney disease requiring renal replacement therapy before study inclusion differed significantly between groups (p = 0.003), being observed in 11.5% of patients in the death group (n = 12) compared with 34.4% among survivors (n = 11). In contrast, the need for renal replacement therapy during hospitalization was highly prevalent in both groups, without a statistically significant difference (55.8% in non-survivors vs. 53.6% in survivors; p = 0.962).

**Table 3:** Univariate comparison between survivors and non-survivors.

**Variable**	**Survivors (%)**	**Deaths (%)**	**p-value**
	**n=32**	**n=104**	
Age (mean ± SD)	57.3 ± 16	64 ± 15.4	**0.042**
ICU admission required	22 (68.8%)	97 (93.3%)	**<0.001**
Length of hospital stay (mean ± SD)	49±40.2	28.9 ± 21.2	**0.010**
**Comorbidities**			
Pre-existing heart disease	7 (21.9%)	13 (12.5%)	0.190
Diabetes mellitus	9 (28.1%)	36 (34.6%)	0.495
Chronic lung disease	1 (3.1%)	6 (5.8%)	0.554
Chronic kidney disease	13 (40.6%)	18 (17.3%)	**0.006**
Immunosuppression	9 (28.1%)	41 (39.4%)	0.246
COVID-19	3 (9.4%)	13 (12.5%)	0.631
Oncological disease	9 (28.1%)	40 (38.5%)	0.287
Exclusive palliative care	1 (3.1%)	20 (19.2%)	**0.027**
Previous *Candida* colonization	4 (12.5%)	26 (25%)	0.136
**Candidemia staging evaluation**			
Fundoscopy performed	10 (31.3%)	15 (14.4%)	**0.032**
Echocardiography performed	22 (68.8%)	27 (26%)	**<0.001**
Abdominal CT scan	1 (3.1%)	3 (2.9%)	0.944
Follow-up blood cultures performed	20 (62.5%)	30 (28.8%)	**<0.001**
Abnormal staging findings	3 (12.5%)	6 (9%)	0.618
Deep focus identified	5 (17.9%)	6 (8.1%)	0.157
Persistent candidemia	1 (3.1%)	5 (4.8%)	0.685
**Adherence to the care bundle**	3.66±2.47	1.98±2,34	**<0.001**
**Bundle score**			**<0.001**
No bundle items	5 (15.6)	42 (40.4)	
1–4 bundle items	9 (28.1)	42 (40.4)	
5–8 bundle items	18 (56.3)	20 (19.2)	
**Species isolated in first blood culture**			
*Candida albicans*	7 (21.9%)	37 (35.6%)	0.147
*Candida parapsilosis complex*	17 (53.1%)	23 (22.1%)	**<0.001**
*Candida glabrata*	2 (6.3%)	16 (15.4%)	0.182
*Candida tropicalis*	2 (6.3%)	18 (17.3%)	0.122
Antifungal treatment duration (mean ± SD)	14.1 ± 16.5	11.5 ± 14.2	0.560
Sequential blood cultures collected within 48–72h	9 (28.1%)	8 (7.7%)	**0.002**
Hypotension (MAP < 65 mmHg)	10 (32.2%)	81 (78.6%)	**<0.001**
**Vasopressor use**			**0.001**
Norepinephrine	8 (25%)	79 (76%)	**0.001**
Vasopressin	2 (6.3%)	54 (51.9%)	**0.001**
Invasive mechanical ventilation	10 (32.2%)	83 (79.8%)	**<0.001**
Vascular catheter present	27 (84.4%)	96 (92.3%)	0.182
Short-term central venous catheter	15 (46.9%)	80 (76.9%)	**0.001**
Temporary hemodialysis catheter	7 (21.9%)	36 (34.6%)	0.175
Tunneled long-term dialysis catheter	8 (25%)	6 (5.8%)	**0.002**
Totally implantable venous access device	1 (3.1%)	2 (1.9%)	0.686
**Catheter removed**	21 (84%)	36 (38%)	**<0.001**
Late catheter removal	9 (28.1%)	10 (9.7%)	**0.009**
Parenteral nutrition	2 (6.3%)	16 (15.4%)	0.182
Renal replacement therapy during hospitalization	18 (53.6%)	58 (55.8%)	0.962
Pre-existing dialysis dependency	11 (34.4%)	12 (11.5%)	**0.003**
Received antifungal therapy	23 (71.9%)	47 (45.2%)	**0.008**
Empirical antifungal therapy	1 (4.3%)	2 (4.2%)	0.972
Step-down (sequential) therapy	5 (22.7%)	4 (8.5%)	0.102
**Antifungal agents used**			
Fluconazole	17 (53.1%)	26 (25%)	**0.003**
Micafungin	14 (43.8%)	34 (32.7%)	0.252
Amphotericin B deoxycholate	0 (0%)	2 (1.9%)	0.429
Liposomal amphotericin B	1 (3.1%)	0 (0%)	0.070
Optimal treatment	14 (43.8%)	24 (23.1%)	**0.023**
SOFA score (mean ± SD)	6.22±2.86	8.95±4.71	0.059
Time from admission to candidemia (mean ± SD)	21.41±21.65	18.15±16.9	0.473
Time from surgery to candidemia (mean ± SD)	19.40±60.22	18.15±16.9	0.228
Time to negative blood culture (mean ± SD)	13.8 ± 12.84	8.97±5.52	0.145
Time to catheter removal (mean ± SD)	9.39±9.89	6.00±6.88	0.211
Time to start antifungal therapy (mean ± SD)	7.95±5.59	4.66±2.56	0.014
Catheter-only blood culture positivity	6 (18.8%)	22 (21.2%)	0.769
C-reactive protein (CRP)	12.7 ± 11.9	22.2 ± 13.4	**0.003**
Previous *Candida* colonization	5 (15.6%)	27 (26%)	0.228
Broad-spectrum antibiotic use	29 (93.5%)	97 (94.2%)	0.897
Presumed bacterial infection	18 (56.3%)	80 (76.9%)	**0.023**
Infectious Diseases consultation	16 (50%)	30 (28.8%)	**0.027**
Abdominal surgery	4 (19%)	14 (25%)	0.583
C-reactive protein (CRP) (mean ± SD)	12.7 ± 11.9	22.2 ± 13.4	**0.003**

ICU, Intensive Care Unit; SD, Standard Deviation; MAP, Mean Arterial Pressure; CRP, C-Reactive Protein; SOFA, Sequential Organ Failure Assessment; CT, Computed Tomography.

Overall adherence to the candidemia care bundle in the total cohort showed a mean of 2.38 implemented items (SD ±2.47). When management strategies were compared according to outcome, survivors received care more closely aligned with guideline recommendations, with a mean of 3.66 bundle items performed (SD ±2.47), compared with 1.98-items (SD ±2.34) among non-survivors (p = 0.001). Score category analysis further demonstrated this disparity (p < 0.001): 56.3% of survivors received the majority of recommended interventions (5–8 items), whereas only 19.2% of patients in the death group reached this level of adherence (Table 3).

**Fig. 2:**
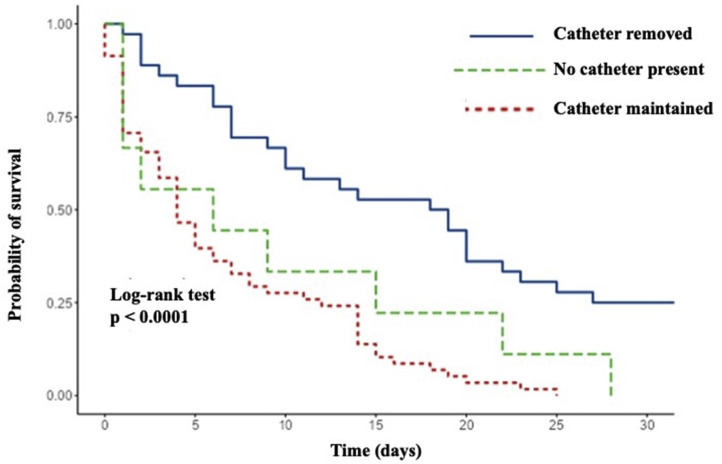
Kaplan-Meier survival curves according to catheter management. Log-rank test, p < 0.0001.

In multivariable logistic regression analysis, lack of optimal treatment emerged as an independent determinant of in-hospital mortality (OR = 5.52, 95% CI 1.17–26.13; p = 0.031), followed by failure to remove the central venous catheter, which significantly increased the odds of death (OR = 6.60, 95% CI 1.84–23.65; p = 0.004). ICU admission also remained independently associated with outcome (OR = 0.08, 95% CI 0.01–0.59; p = 0.013) (Table 4). Supporting these findings, Kaplan-Meier survival analysis demonstrated significant differences in survival according to catheter management strategy (log-rank p < 0.001) (Fig. 2).

**Table 4:** Multivariable logistic regression analysis of factors independently associated with in-hospital mortality.

**Predictor**	**Adjusted OR**	**95% CI**	**p-value**
ICU admission	0.08	0.01–0.58	**0.013**
Dialysis-dependent CKD	6.22	0.75–51.69	0.091
*Candida parapsilosis* complex	0.52	0.13–2.05	0.346
Vasoactive drug use	0.52	0.15–1.82	0.308
No catheter removal	6.60	1.84–23.65	**0.004**
Non-optimal treatment	5.52	1.17–26.13	**0.031**
Presumed bacterial infection	1.18	0.25–5.64	0.831
No infectious diseases evaluation	0.90	0.23–3.43	0.872

ICU, intensive care unit; CKD, chronic kidney disease; OR, odds ratio; CI, confidence interval.

## Discussion

The analysis of candidemia episodes in this cohort revealed an extremely high in-hospital mortality rate, substantially exceeding figures reported in contemporary series from high-income countries and even surpassing most Brazilian cohorts published in the last decade. This finding cannot be interpreted solely as a reflection of the intrinsic severity of the patients, characterized by the high need for ICU admission, mechanical ventilation, and vasopressor support, but also as an indication of weaknesses in early recognition, source control, and systematic adherence to recommended candidemia management measures. The predominance of critically ill patients, the low implementation of the care bundle, and the limited participation of the Infectious Diseases team describe a scenario in which candidemia behaves not only as a marker of severity, but as a clinical event whose outcome is strongly influenced by the quality of care provided, including non-adherence to recommended management strategies. In this context, the results of this study suggest that the high observed lethality is less related to the microbiological profile of the isolated species and more directly associated with failures in modifiable interventions, particularly early vascular source control and adherence to evidence-based care recommendations.

Regarding baseline characteristics, advanced age and vasopressor requirement were associated with mortality in univariate analysis, however, these variables did not retain significance in the multivariable model, suggesting that their effect is likely mediated by overall disease severity rather than acting as independent predictors. Previous studies have consistently identified advanced age and hemodynamic instability as important determinants of poor outcomes in candidemia,^5^, ^6^, ^7^, ^8^, ^9^, ^10^, ^11^ although our adjusted analysis did not confirm these associations as independent predictors. Sex and ethnicity were not associated with outcomes in this cohort. Pre-existing dialysis-dependent chronic kidney disease was more frequent among survivors in univariate analysis but was not confirmed after adjustment, in contrast to prior studies that have identified renal failure and hemodialysis as risk factors for mortality.^5^^,^^10^ Other comorbidities, including diabetes mellitus and solid malignancies, were not associated with mortality, although their high prevalence is consistent with previous reports,^5^^,^^6^ reflecting the substantial burden of chronic disease among patients with candidemia in tertiary-care settings.

Epidemiologically, the species distribution in this cohort reflects the pattern described by Hamburger et al. (2024)[4] for middle-income countries, with non-albicans species accounting for 67.6% of isolates. *C. albicans* represented only 32.4% of cases, similar to rates observed in oncological cohorts (Leal de Almeida et al., 2024), but lower than those reported in general Brazilian ICUs, where it remains predominant (43.6%).^6^^,^^7^ Internationally, a rise in *C. glabrata* has been documented in the United Kingdom and the United States,^9^^,^^12^ whereas in this study the *C. parapsilosis* complex predominated among non-*albicans* species (21.3%), followed by *C. tropicalis* (14.7%), consistent with traditional Brazilian fungal ecology.^7^ Agnelli et al. (2022)[5] found that infection caused by species of the *C. parapsilosis* complex is associated with more favorable outcomes due to its lower virulence. In our cohort, although this association was observed in univariate analysis, it did not remain significant after multivariable adjustment. However, these findings should be interpreted with caution, as antifungal susceptibility testing was practically not performed in this cohort, precluding the assessment of resistance patterns and their impact on clinical outcomes. In contrast, failure to remove the central venous catheter remained an independent predictor of mortality, increasing the risk of death by 6.60-fold (p = 0.004), consistent with the findings of Logan et al. (2026)[12] and current international guideline recommendations.^13^^,^^14^ Therefore, antifungal strategies supported by current evidence achieve optimal effectiveness only when combined with adequate source control and awareness of local epidemiology.^15^

Mejia et al. (2019)^16^ demonstrated a positive impact of Infectious Diseases consultation on outcomes in candidemia. In our cohort, Infectious Diseases consultation was also associated with improved survival in univariate analysis, as only 28.8% of patients in the death group were evaluated (p = 0.027), however, this association did not retain significance in the multivariable model. Nevertheless, certain aspects of management were noted in this subgroup. Among the nine patients who underwent step-down therapy, eight had been evaluated by Infectious Diseases. Of the four deaths in this subgroup, one patient had not received specialist consultation, and all patients survived for more than 15-days after candidemia diagnosis. A similar pattern was observed among patients with deep foci of infection, in whom most received specialist consultation, and mortality tended to occur later. Additionally, effective source control was more frequently achieved in patients evaluated by Infectious Diseases. These findings suggest that Infectious Diseases involvement may facilitate the implementation of complex interventions and continuity of care, although its independent effect could not be confirmed after adjustment.

Antifungal therapy was initiated in only 51.5% of patients, representing a concerning gap in care that appears even greater than that reported in previous Brazilian studies,^5^, ^6^, ^7^, ^8^ where treatment initiation rates are generally higher, underscoring missed opportunities for early therapeutic intervention. This tertiary center exhibited an in-hospital mortality rate of 76.47%, markedly higher than recent international cohorts, reporting lethality between 25% and 40%.^5^, ^6^, ^7^^,^^9^^,^^12^^,^^11^^,^^17^ This discrepancy reflects the critical severity of the studied population, composed predominantly of patients under advanced life support, with 79.8% requiring mechanical ventilation and 76.7% requiring vasopressor support in the death group. In the national context, this finding corroborates the critical stagnation described by Dalla Lana et al. (2020),^18^ demonstrating that candidemia mortality in Brazil has remained above 50% for two decades.

Low adherence to management protocols emerged as an explanatory variable for unfavorable outcomes. In this study, optimal treatment was achieved in only 27.9% of cases, and among those who died, only 23.1% reached this target (p = 0.023). Absence of optimal treatment was consistently associated with increased mortality in both univariate and multivariable analyses, highlighting adherence to guideline-based management bundles as a key modifiable determinant of outcome in candidemia. This gap in care is corroborated by the multinational European ECMM *Candida* III study,^19^ which demonstrated a direct association between high guideline adherence and improved survival at 7- and 30-days. The underuse of staging examinations, such as echocardiography, performed in only 26% of patients who died (p < 0.001), contrasts with international recommendations.^13^^,^^14^ Araujo et al. (2024)^20^ reported high adherence rates, such as catheter removal (82.5%) and follow-up blood cultures (91.2%), contrasting with the present study, which showed a mean bundle adherence of only 2.38-items. Despite this quantitative disparity, both studies highlight the potential importance of adherence to management protocols in candidemia care.

Improvement in candidemia management at the institutional level should begin with strengthening diagnostic stewardship, particularly timely and adequate blood culture collection in patients with suspected bloodstream infection. This includes reinforcing appropriate sampling practices, obtaining follow-up blood cultures after candidemia diagnosis, and ensuring rapid communication of positive results to the clinical team. These measures should be integrated with prompt antifungal initiation, early vascular source control, and systematic Infectious Diseases consultation. The implementation of structured institutional protocols and care bundles may represent a key strategy to improve adherence to recommended management and, consequently, patient outcomes.

This study has several limitations that should be considered when interpreting its findings. First, its retrospective, single-center design relies on the accuracy and completeness of medical records, potentially introducing information bias and limiting external validity. Additionally, a proportion of eligible patients was excluded due to unavailable clinical data, precluding a detailed comparison of baseline characteristics, temporal distribution, and hospital unit allocation between included and non-included cases, which may introduce potential selection bias. The results reflect the local fungal ecology, case-mix severity, and institutional practices of a tertiary referral center, which may not be generalizable to other epidemiological or healthcare settings. Race/ethnicity data were extracted from administrative records rather than self-reported information, introducing the possibility of heteroattribution bias. The candidemia care bundle was analyzed as an unweighted composite measure of adherence; the use of validated and weighted tools, such as the EQUAL *Candida* score, may provide a more standardized and granular assessment of guideline adherence. Incomplete staging investigations, including limited performance of fundoscopy, echocardiography, serial blood cultures, and antifungal susceptibility testing, may have led to underestimation of deep-seated or complicated candidiasis. The study did not systematically evaluate time-to-intervention variables, such as time to antifungal initiation or catheter removal, which could influence mortality outcomes. Although multivariable analysis was performed, the relatively modest sample size may have limited statistical power, and residual confounding cannot be fully excluded. In particular, confounding by indication may have influenced associations related to vasopressor use and catheter management decisions. Additionally, candidemia was not stratified according to primary versus secondary source, and long-term post-discharge outcomes were not assessed. Finally, although cases with catheter-only positive blood cultures were included, the comparable severity indicators and clinical outcomes between groups mitigate, but do not completely eliminate, the possibility of misclassification bias.

## Conclusion

Taken together, these findings suggest that candidemia mortality in this setting is not merely an inevitable consequence of critical illness but is closely linked to non-adherence to recommended management strategies, particularly delayed source control and suboptimal treatment. This underscores quality of care and adherence to evidence-based practices as central and potentially modifiable determinants of outcome.

## Ethics approval and consent to participate

The study was conducted in accordance with the Declaration of Helsinki, and approved by the Institutional Review Board of the Centro Universitário FMABC (CAAE 89896925.1.0000.0082) and authorized by the HEMC clinical directorate.

## Informed consent statement

Patient consent was waived due to the design of study (retrospective, based on analyses of clinical and environmental samples).

## Consent for publication

All authors have read and agreed to the published version of the manuscript.

## Data availability

Not applicable.

## Funding

The authors declare no funding.

## CRediT authorship contribution statement

**Murilo Freua Sequeira:** Investigation, Conceptualization, Data curation, Writing – original draft, Writing – review & editing. **Caio Trevelin Sambo:** Investigation, Conceptualization, Data curation. **Bianca Leal de Almeida:** Conceptualization. **Erika Yukie Ishigaki:** Investigation, Data curation. **Eloisa Basile Siqueira Ayub:** Investigation, Conceptualization. **Gabriel Fialkovitz:** Conceptualization. **Adriana Satie Gonçalves Kono Magri:** Writing – original draft. **Olavo Henrique Munhoz Leite:** Investigation, Conceptualization, Writing – review & editing. **Marcello Mihailenko Chaves Magri:** Conceptualization, Supervision, Writing – review & editing, Writing – original draft.

## Conflicts of interest

M.M.C.M has received support for attending educational meetings from Knight and Mundipharma. The remaining authors declare no conflict of interest.
